# Methylmercury and IQ: Dose–Response Estimate of Prenatal Effect

**Published:** 2007-04

**Authors:** Valerie J. Brown

Methylmercury, the most biologically active mercury compound, is well known to cause serious health effects, particularly to the developing fetal nervous system. Effects can include attention deficits as well as IQ, motor, memory, and language impairment. A new analysis now combines data from three earlier studies to produce an integrated estimate of the dose–response relationship between maternal mercury exposure during pregnancy and lowered childhood IQ **[*EHP* 115:609–615; Axelrad et al.]**.

The authors analyzed combined IQ data from three longitudinal studies conducted in the Faroe Islands, the Seychelles Islands, and New Zealand. These studies measured a variety of neurodevelopmental end points, including IQ, attention, and motor skills. The range of pre-natal exposures in the three populations is comparable to those of some U.S. populations. For example, a 2003 study found the lowest maternal blood mercury level in the Faroe Islands to be 0.53 μg/L, and the CDC reported in 2004 that more than half of U.S. women had blood mercury concentrations higher than this. Geometric mean blood concentrations in the United States from 1999 to 2002 were 0.92 μg/L for women of childbearing age; for children the mean was 0.33 μg/L.

The New Zealand and Seychelles studies reported results in terms of ppm of hair mercury, whereas the Faroe Islands study reported effects in terms of ppb of cord blood mercury. So the team converted the Faroe Islands results to their equivalents in units of hair mercury. They found a childhood IQ decrease of 0.18 points for each ppm rise in maternal hair mercury. The team assumed a linear, nonthreshold dose–response curve. However, they noted that if very low exposures produce a steeper curve, as has been found recently with childhood lead exposure, their calculation may underestimate the effects of prenatal mercury exposure. Similarly, certain cognitive abilities such as word retrieval and retention of verbally presented information are not captured by IQ scores, so relying only on IQ as a measure of cognitive function will also underestimate mercury’s effects.

Eating fish is the most common route of human exposure to methylmercury. In 2004 the FDA and the EPA issued a joint statement advising women of childbearing age and children to limit their weekly consumption of commercially caught fish to 12 oz (6 oz for locally caught fish) in order to avoid harmful exposure. The EPA has set a reference dose of 0.1 μg/kg/day for methylmercury as an estimate of the daily exposure unlikely to cause harm over a lifetime.

Methylmercury’s effect on IQ is separate from its effect on attention and motor skills. But because IQ is a well-established end point used in cost–benefit and economic analyses of the effects of environmental contaminants, establishing the dose–response relationship for IQ is a first step in quantifying the benefits of reducing mercury exposure.

## Figures and Tables

**Figure:**
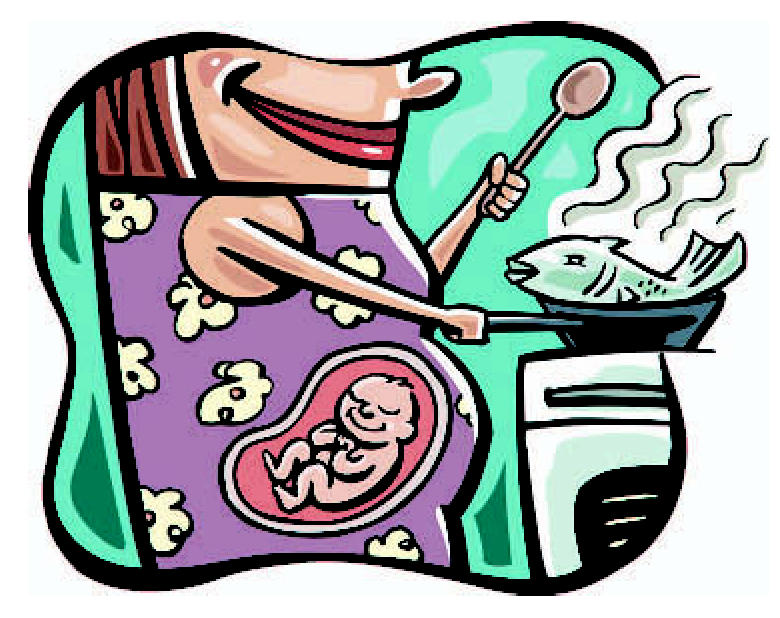
Smart move A new analysis takes a first step toward quantifying the benefits of reducing mercury exposure, which may include avoiding IQ deficits.

